# Sequali: efficient and comprehensive quality control of short- and long-read sequencing data

**DOI:** 10.1093/bioadv/vbaf010

**Published:** 2025-01-29

**Authors:** Ruben H P Vorderman

**Affiliations:** Sequencing Analysis Support Core, Department of Biomedical Data Sciences, Leiden University Medical Center, Leiden 2300RC, Netherlands

## Abstract

**Motivation:**

Quality control of sequencing data is the first step in many sequencing workflows. Short- and long-read sequencing technologies have many commonalities with regard to quality control. Several quality control programs exist; however, none possess a feature set that is adequate for both technologies. Quality control programs aimed at Oxford Nanopore Technologies sequencing lack vital features, such as adapter searching, overrepresented sequence analysis, and duplication analysis.

**Results:**

Sequali was developed to provide sequencing quality control for both short- and long-read sequencing technologies. It features adapter search, overrepresented sequence analysis, and duplication analysis and supports FASTQ and uBAM inputs. It is significantly faster than comparable sequencing quality control programs for both short- and long-read sequencing technologies.

**Availability and Implementation:**

Sequali is an open-source Python application using C extensions and is freely available under the AGPL-3.0 license at https://github.com/rhpvorderman/sequali. The source code for each release is archived at zenodo: https://zenodo.org/doi/10.5281/zenodo.10822485.

## 1 Introduction

Quality control of sequencing data is the first step in many sequencing workflows. Though many different sequencing technologies are available, there are many commonalities between platforms with regard to quality control. Statistics pertaining to sequence length, sequence composition, and sequence quality are relevant for all platforms. Furthermore, both long- and short-read sequencing technologies use adapter sequences and barcodes, which makes sequence content analysis necessary to determine whether these helper sequences are still present in the library. Duplication profiles are useful in both PCR and PCR-free methods as the rate of natural read duplication differs between DNA and RNA experiments (Bansal 2017).

Nanopore sequencing technology enables a wide variety of applications (Pugh 2023). Since the launch of the MinION device in 2015, various tools have been launched to assess the quality of Oxford Nanopore Technologies (ONT) sequencing data, such as NanoPlot (De Coster and Rademakers 2023), MinIONQC (Lanfear *et al.* 2018), and pycoQC (Leger and Leonardi 2019). These tools work from sequencing summary data and, as such, do not report on sequence composition and sequence content, nor do they provide information on the duplication profile. As a result, these tools may miss problematic libraries with adapter and barcode contaminations. Channel translocation speed deviations are an important source of insertions and deletions (Delahaye and Nicolas 2021), but NanoPlot, MinIONQC, and pycoQC do not provide any plots on translocation speed.

FastQC (Andrews 2010) and Fastp (Chen *et al.* 2018) are commonly used for short-read sequencing and do provide statistics for adapter and barcode contaminations. However, FastQC and Fastp do not provide proper support for ONT data. ONT adapters are not included by default, and the Q scores in their quality metrics are not based on expected error rate, as is best practice (Edgar and Flyvbjerg 2015). As a result, the average quality of reads is overestimated. Not using expected error rates to calculate the quality also limits the viability of FastQC and Fastp for short-read sequencing data. This was brought to the attention of the developers in 2023, but not fixed at the time of writing this paper.

In order to address these concerns, this paper presents a new tool for sequencing quality control: Sequali.

## 2 Implementation

Sequali is a collection of sequence analysis modules written in C with Python bindings. The Sequali application is written in Python and handles the overarching logic as well as the report generation. It has been made available on the Python Package Index (PyPI), Bioconda (Grüning *et al.* 2018), and BioContainers (Da Veiga Leprevost *et al.* 2017).

**Figure 1.**
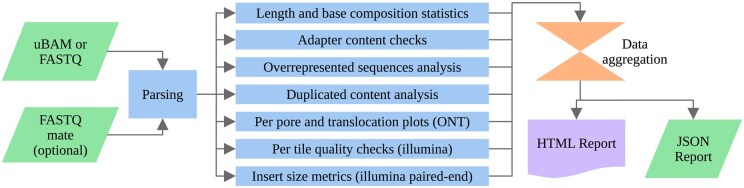
Flowchart representation of Sequali’s internal workflow.

One module handles generic statistics, such as Phred score and base content. The adapter content module searches for small 12 bp probes for single-end data and uses a fast multiple pattern-matching algorithm (Mukherjee 2010). For paired-end, data read overlap is used to determine the adapter and insert size as this provides better detection (Turner 2014). The duplication estimation module takes a small 16 bp fingerprint from the read and uses the technique described by Xie *et al.* (2013) to gain an accurate estimate of the duplicated content. To check for adapter and helper sequences that are not included in the adapter search module, one in eight reads is sampled into small fragments. The first 5 million fragments are stored, and their abundance is counted in the rest of the sample. Any fragment with a higher than 0.1% occurrence in the sample is identified against the NCBI UniVEC database (NCBI 2023) and some known ONT barcodes and adapters, which are not included in UniVec yet. The identification occurs by gathering candidates using canonical k-mers and determining sequence identity using Smith–Waterman alignment. Separate modules for Illumina (per tile quality, insert size metrics) and ONT sequencing (per channel plots, translocation speed plots) are provided. A flowchart of Sequali’s internal workflow is shown in Fig. 1.

Sequali offers MultiQC (Ewels *et al.* 2016) integration and works from Sanger FASTQ (Cock *et al.* 2010) and uBAM (Li *et al.* 2009) formats.

To improve the speed of the application, the Python bindings for the ISA-L (Tucker *et al.* 2017) library are used to speed up decompression speed of the input files.

## 3 Methods

Only tools that can work from FASTQ data and were still actively maintained were considered for comparison. NanoPlot, FastQC, and Fastp are all popular tools that can handle the FASTQ data format. Falco is a C++ reimplementation of FastQC that vastly improves the runtime characteristics (de Sena Brandine and Smith 2019). Since Falco and FastQC effectively create the same outputs, Falco is used in the performance comparison. Since Fastp is actually also a read modification tool, all its modification options are disabled using the -A -G -Q -L flags as was done in the Falco paper (de Sena Brandine and Smith 2019). Furthermore, despite disabling all read modification options, Fastp still runs a before and after modification reporting. Fastp runtime is divided by two to correct for it running the QC twice. The Fastp runtime performance is greatly affected by turning on overrepresented sequence sampling, so it is run both with and without.

For comparing the ONT data performance, the ERR3988483 dataset from a study by Shafin *et al.* (2020) was used. For comparing performance on Illumina reads, the ERR11204024 dataset was used containing two paired-end FASTQ files. For tools that cannot natively handle paired-end data, the tool was run on each file, and the run times were added. Links to the benchmark files are provided on the Sequali GitHub page.

Runtime was measured by adding reported system time and user time as reported by the GNU time (GNU 2018) application. Tools were run with apptainer 1.2.5 with the—containall flag using a container provided by BioContainers (Da Veiga Leprevost *et al.* 2017) with only the test data directory mounted on a AMD Ryzen 5 3600 system with 2x16GB DDR4-3200 memory.

## 4 Results

An overview of the feature comparison is shown in Table 1. Sequali and NanoPlot both use the expected error rate to calculate the average Phred score, whilst FastQC/Falco and Fastp average the Phred scores. As a result, FastQC/Falco and Fastp consistently report much higher quality scores than NanoPlot and Sequali. For example, Falco reports over 1 000 000 reads in the ERR3988483 file to be at quality Q ≥ 20 and more than 700 000 reads to be at quality Q ≥ 30. In contrast, Sequali and NanoPlot report just 28 reads to be Q ≥ 15.

**Table 1.** Feature table.

	Sequali	FastQC/Falco	Fastp	NanoPlot
Length plots	Yes	Yes	Yes	Yes
Phred score plots	Yes	Yes, but overestimates quality	Yes, but overestimates quality	Yes
GC content plots	Yes	Yes	Yes	No
Per read averages	Yes	Yes	No	Yes
ONT channel plots	Yes	No	No	Yes
ONT translocation speed plot	Yes	No	No	No
Illumina per tile quality plot	Yes	Yes	No	No
Insert size metrics	Yes	No	Yes	No
Adapter content checks	Illumina, ONT	Illumina	Illumina, MGI/BGI	None
Overrepresented sequences check	Against UniVec database and ONT barcodes (> 6000 sequences)	Custom database (> 150 sequences)	Yes, but sequences are not identified.	No
Sequence duplication profile	Yes	Yes, slightly overestimates unique reads.	No, estimates total duplicates only	No, does not estimate

FastQC, Fastp, and Sequali perform similar overrepresented sequence analyses where the first reads are sampled, and the samples are checked for occurrence in the other reads. FastQC takes only a sample from the beginning of the read, while Fastp and Sequali can sample across the entire read. Fastp stores the indices at which these samples commonly occur. This more thorough check leads to increased runtime, and the check is turned off by default. FastQC and Sequali both check overrepresented sequences against a database. All tools use subsampling to reduce the computational load.

FastQC provides slightly inaccurate estimates for duplication because taking the first 100 000 reads and counting their occurrences tends to overestimate unique reads (Xie *et al.* 2013). Fastp uses a bloom filter and cannot count if sequences are duplicated multiple times, therefore, omitting a duplication profile plot. Sequali uses hash-based sampling resulting in an accurate estimate whilst using little memory (Xie *et al.* 2013).

The results of the performance comparison are shown in Table 2. Sequali is the fastest QC program of the programs tested. Most programs have low memory usage, though Fastp does not scale well to ultra-long reads, requiring over 30 GB to finish the run.

**Table 2.** Performance comparison.

	Sequali	FastQC/Falco	Fastp	NanoPlot
Biocontainer tag	11.1—py312hf67a6ed_0	2.2—hdcf5f25_0 (Falco)	0.23.4—h125f33a_5	1.43.0—pyhdfd78af_0
Runtime ERR11204024	0m19s	0m35s	0m26s/0m52s^a^^,^^b^	2m41s
Runtime ERR3988483	3m22s	17m12s	8m39s/11m30s^a^^,^^b^	23m28s
Memory usage ERR11204024	0.5G	0.1G	1.8G/2.2G^a^	1.0G
Memory usage ERR3988483	0.7G	0.6G	30.7G/30.9G^a^	1.4G

a Without/with overrepresented sequences analysis enabled.

b FastP runtimes are divided by two to account for the fact that it runs the quality control modules twice.

## 5 Discussion

For short-read sequencing, Sequali offers an improved experience over FastQC/Falco with faster runtimes, a more extensive overrepresented sequences check that also checks the ends of reads, Phred averaging based on expected error rate, and paired-end support with insert size metrics. Fastp offers a suite of FASTQ manipulation tools, which Sequali does not offer. However, running only the QC component, Sequali is much faster whilst offering very useful statistics such as per read average quality and per read GC content plots.

Sequali offers significantly more features than ONT-oriented tools. Currently, none of the mentioned ONT-oriented tools do any analysis based on the content of the sequence. While FastQC/Falco and Fastp can technically also be used with ONT data, Sequali offers better integration by having adapter and bar code content searches for ONT-specific sequences, specific plots for channel usage, and translocation speed as well as much lower resource requirements for long-read data.

Due to its suitability for both long- and short-read data, Sequali can be used as a universal QC tool, replacing the need for having separate QC tools for ONT and Illumina data.

## Data Availability

Sequali is freely available under the AGPL-3.0 license. The source code and additional examples can be found at https://github.com/rhpvorderman/sequali. The source code for each release is archived at zenodo: https://zenodo.org/doi/10.5281/zenodo.10822485. Binary releases for Linux, Windows and macOS are available on PyPI (https://pypi.org/project/sequali/). Binary releases for Linux and macOS are available on Bioconda (https://bioconda.github.io/recipes/sequali/README.html). Installation can be via pip, conda or by using a container provided by the BioContainers project.
